# Supplementary material for: Advanced Nursing Practice in the context of Primary Health Care in Brazil: documentary research
Source: Rev Bras Enferm. 2025 Dec 8;78(Suppl 4):e20250155. doi: 10.1590/0034-7167-2025-0155 (PMC12700577; doi:10.1590/0034-7167-2025-0155)
Supplement: Translation

# Translation: Prática Avançada de Enfermagem no âmbito da Atenção Primária à Saúde no Brasil: pesquisa documental

**DOI:** 10.1590/0034-7167-2025-0155pt

**Authors:** Emerson Willian Santos de Almeida, Italo Rodolfo Silva, Simone de Godoy, Beatriz Rosana Gonçalves de Oliveira Toso, Elizimara Ferreira Siqueira, Ellen Marcia Peres, Jeanne-Marie Rodrigues Stacciarini, Isabel Amélia Costa Mendes

**Affiliations:** I Universidade de São Paulo. Ribeirão Preto, São Paulo, Brasil; II Universidade Federal do Rio de Janeiro. Macaé, Rio de Janeiro, Brasil; III Universidade Estadual do Oeste do Paraná. Cascavel, Paraná, Brasil; IV Prefeitura Municipal de Florianópolis. Florianópolis, Santa Catarina, Brasil; V Universidade Estadual do Rio de Janeiro. Rio de Janeiro, Rio de Janeiro, Brasil; VI University of Michigan. Ann Arbor, Michigan, Estados Unidos da América

**Keywords:** Prática Avançada de Enfermagem, Atenção Primária à, Saúde, Sistema Único de Saúde, Enfermagem, Política de Saúde.

## Abstract

**Objetivos:**

categorizar atividades de Práticas Avançadas de Enfermagem no Brasil registradas na plataforma de relatórios de Saúde/Produção do Sistema de Informação em Saúde para a Atenção Básica por enfermeiros da Atenção Primária à Saúde.

**Métodos:**

estudo documental, exploratório e de abrangência nacional, conduzido em 2024, com base em dados públicos disponíveis em sites vinculados ao Ministério da Saúde no Brasil, ano de referência 2023.

**Resultados:**

a matriz de análise representa uma estrutura conceitual comparativa e demonstrativa da evidência de práticas avançadas que vêm sendo assumidas e registradas pelos enfermeiros no sistema de dados do Sistema Único de Saúde, com acesso aberto.

**Considerações Finais:**

o estudo evidencia que, apesar da autonomia dos enfermeiros em práticas, como prescrição de medicamentos, solicitação de exames, encaminhamento, inserção de DIU, sutura e outras intervenções, a política brasileira ainda apresenta discrepâncias em relação às diretrizes do *International Council of Nurses* e da Organização Mundial da Saúde.

## INTRODUÇÃO

Fundamentado nos princípios da universalidade, integralidade e equidade, o Sistema Único de Saúde (SUS), sistema de saúde vigente no Brasil, garante assistência à população e tem sido implementado há 35 anos, apresentando fortalezas e desafios^(1)^. Nessa conjuntura, integrados por interações políticas, econômicas e institucionais, corrobora-se o entendimento de que cabe aos sistemas de saúde gerenciar e prover recursos aos seus subsistemas e processos capazes de atender aos propósitos em favor da saúde da população, materializando-se em estruturas, normas e serviços destinados a alcançar resultados alinhados à concepção de saúde no contexto de uma nação. Ademais, sua abrangência envolve organizações, profissionais, serviços, insumos, tecnologias e conhecimentos, cujo objetivo é promover a saúde, prevenir doenças e oferecer cuidados e reabilitação^(2)^.

A relevância do SUS está pautada nos princípios supracitados, mas também em seu modelo de organização. Nesse sentido, está dividido em níveis de atenção capazes de atender, estrategicamente, as complexidades de saúde, de modo a organizar-se em Atenção Primária à Saúde (APS), Atenção Secundária de Saúde e Atenção Terciária. Entretanto, a APS representa a principal esfera para garantir o direito à saúde, especialmente em decorrência de ser, dentre as demais, o contexto responsável por cerca de 80% das demandas de saúde e, por conseguinte, dos processos decisórios que envolvem os cuidados com a saúde da população, de modo a reduzir o fluxo de adoecimentos e agravos dos pacientes destinados à Atenção Terciária, sobretudo no que diz respeito aos processos cirúrgicos e de internação hospitalar^(3)^.

Nesse sentido, no contexto da Política Nacional de Atenção Básica - PNAB - (Brasil, 2017), tem-se que a APS engloba ações que visam a prevenção, proteção, diagnóstico, tratamento, reabilitação, redução de danos, cuidados paliativos, promoção e vigilância em saúde, desenvolvidas por equipes multiprofissionais^(4)^. Assim, por meio do trabalho desenvolvido por médicos de família, enfermeiros especializados em saúde comunitária, agentes comunitários, além de outros profissionais capacitados, essa abordagem integra competências interdisciplinares e fortalezas intersetoriais para enfrentar os determinantes sociais de saúde. Além de ser resolutiva, a APS intercepta demandas e oferece respostas eficazes, consolidando-se como o modelo ideal para o SUS^(5)^.

Portanto, semelhante a outros sistemas de saúde, o funcionamento do SUS depende, fundamentalmente, da capacidade de promover saúde bem como do atendimento de pessoas adoecidas que demandam cuidados profissionais; desse modo, para ser eficaz, o sistema está subordinado a um corpo profissional qualificado, ao encontro do entendimento de que o seu eixo primordial é o capital humano. A despeito de tal realidade, tem-se que a enfermagem compõe a maior força de trabalho da área da saúde e, por conseguinte, do SUS e dos sistemas de saúde de todas as nações^(3)^.

Instituições internacionais de Enfermagem, como o *International Council of Nurses* (ICN) e órgãos nacionais a ele vinculados, como o Conselho Federal de Enfermagem (COFEN) no Brasil, além de organismos de saúde de abrangência global, como a Organização Mundial da Saúde (OMS), advogam o fortalecimento dos sistemas de saúde, conferindo autonomia profissional ao enfermeiro para qualificar e estender o alcance das práticas assistenciais^(6-11)^. Nessa direção, a Região das Américas, desde 2014, a partir da Organização Panamericana da Saúde (OPS), tem preconizado a Prática Avançada de Enfermagem (PAE) como estratégia para cumprimento de suas resoluções^(10,12,13)^. Essa vertente representa a expansão do escopo da prática profissional, com intervenções baseadas nas melhores evidências, no perfil do paciente, nas competências profissionais, nos recursos tecnológicos e na legislação apropriada que, em conjunto, mediante protocolos robustos, podem ser capazes de produzir eficientes resultados de saúde para indivíduos, famílias e comunidades atendidas por enfermeiros credenciados^(14-17)^.

Considerando a PAE como uma estratégia efetiva para abordar os desafios no acesso, na continuidade e na eficiência da prestação de cuidados primários de saúde, a *Organisation for Economic Co-operation and Development* (OCDE) analisou o seu desenvolvimento em cinco países a ela integrados, os quais se encontram em diferentes estágios de sua implementação: dois países, Estados Unidos e Canadá, que têm uma longa experiência nesse processo, Austrália, que tem uma experiência moderadamente longa, além de França e Itália, que ainda estão na fase inicial de implementação da PAE na APS. A evolução observada no estudo mostra uma crescente disparidade entre os países que lideraram essa prática, nos quais houve uma expansão da PAE, enquanto os que a adotaram apenas recentemente ainda estão em fases iniciais ou ainda debatendo sua adoção. Nos países líderes, essa prática tem demonstrado eficácia na ampliação do acesso à APS, continuidade do cuidado, redução de readmissões hospitalares e maior satisfação dos pacientes, sem prejuízo à qualidade ou à segurança do atendimento, quando devidamente capacitados^(18)^. Importa, contudo, considerar as especificidades, os potenciais e as fortalezas da PAE no maior sistema universal de saúde, notadamente no contexto da APS, a partir do SUS.

Nessa perspectiva, inúmeras e importantes iniciativas têm sido tomadas em termos de intervenções na prática assistencial, nos espaços acadêmicos e nos contextos associativos, regulatórios e de formulação de políticas^(8,19-21)^. Diante do exposto, cumpre questionar: qual o panorama da PAE em alinhamento com o contexto da APS, em se tratando do SUS?

Desse modo, motivados pelo interesse em contribuir para a compreensão da trajetória evolutiva do Enfermeiro de Prática Avançada (EPA) na APS brasileira, propomo-nos a investigar quais são as competências de PAE que os enfermeiros da APS já desenvolvem no país e apresentar evidências que contribuam para a intensificação do debate sobre esse tema e constituam incentivo e subsídio para a implementação da PAE no Brasil.

## OBJETIVOS

Categorizar atividades de Práticas Avançadas de Enfermagem no Brasil registradas na plataforma de relatórios de Saúde/Produção do Sistema de Informação em Saúde para a Atenção Básica por enfermeiros da Atenção Primária à Saúde.

## MÉTODOS

### Aspectos éticos

Este estudo está isento da apreciação do Comitê de Ética em Pesquisa (CEP) vinculado ao Sistema CEP/Conep, uma vez que utiliza exclusivamente dados secundários, sem identificação nominal dos participantes. Os dados analisados são de domínio público e irrestrito, não havendo implicações relacionadas à privacidade, segurança ou ao controle de acesso.

### Desenho, período e local do estudo

Trata-se de um estudo documental^(22)^, exploratório e de abrangência nacional, conduzido em 2024, com base em dados públicos disponíveis em sites vinculados ao Ministério da Saúde no Brasil, ano de referência 2023^(23-25)^.

### Fonte dos dados

Os dados foram extraídos da Plataforma de Relatórios de Saúde/Produção do Sistema de Informação em Saúde para a Atenção Básica (SISAB)^(23)^. No contexto brasileiro, a Política Nacional de Atenção Básica (PNAB) foi restabelecida em 2017, conferindo diretrizes para a organização da Atenção Básica (AB), um dos componentes da Rede de Atenção à Saúde. O documento equipara terminologicamente a AB à APS definida como um conjunto de ações de saúde individuais, familiares e coletivas, abrangendo promoção, prevenção, proteção, diagnóstico, tratamento, reabilitação, redução de danos, cuidados paliativos e vigilância em saúde, conforme disposto no Artigo 2º da PNAB^(4)^.

A PNAB define, nos artigos 6º e 7º, bem como em seu Capítulo I e anexos, as responsabilidades comuns aos profissionais de saúde na APS. Especificamente, o item 4.2.1 detalha as atribuições do enfermeiro. Ademais, o SISAB é um sistema público utilizado para gestão da informação no SUS, permitindo a coleta de dados de produção dos profissionais da APS. O sistema captura informações inseridas via Coleta de Dados Simplificados, Prontuário Eletrônico do Cidadão e aplicativos móveis, como o e-SUS Território e Atividade Coletiva.

Os dados do SISAB refletem as ações realizadas pelos enfermeiros e demais profissionais na APS. Os municípios enviam relatórios mensais ao sistema contendo dados de produção e atividades coletivas. Esse processo assegura que as práticas de enfermagem estejam alinhadas às diretrizes da PNAB.

### Coleta de dados

Os dados foram coletados entre os dias 23 e 27 de junho de 2024, seguindo um protocolo previamente elaborado pelos autores. Esse protocolo compreendeu etapas sistemáticas para garantir a integridade e reprodutibilidade da coleta.

O ICN define vinte e uma características do Enfermeiro de Prática Avançada, distribuídas em três domínios: Preparação Educacional, Natureza da Prática e Exercício de Enfermagem de Prática Avançada. Utilizou-se esse documento norteador global de enfermagem para relacionar as características da enfermagem de prática avançada convergentes com as competências do enfermeiro estabelecidas pela PNAB, segundo as diretrizes para a organização da Atenção Básica, no âmbito do SUS.

Dessa forma, para cada característica preconizada pelo ICN, foi identificada a correspondência com as diretrizes estabelecidas na PNAB, em seu Capítulo I e anexos. Assim, procedeu-se à análise integral da referida política, considerando as principais responsabilidades atribuídas ao enfermeiro no âmbito da APS.

O SISAB reúne as ações específicas realizadas pelos enfermeiros e demais profissionais no âmbito da atenção básica. Os registros são processados pelos municípios, que enviam relatórios mensais ao SISAB, como dados de produção e atividades coletivas. Esse processo assegura que as práticas dos enfermeiros sejam alinhadas às diretrizes da Política Nacional de Atenção Básica (PNAB).

Dessa forma, por meio da extração de dados do SISAB, foram obtidas informações de produção dos enfermeiros e relatórios de indicadores por estado, município, região de saúde e equipe. Para identificar e categorizar as ações específicas realizadas pelos enfermeiros no âmbito da AB, foram mapeados os códigos presentes na plataforma de relatórios do SISAB, nas categorias Saúde/Produção e Saúde/Atividade Coletiva.

Foram considerados procedimentos e atendimentos existentes no Sistema de Gerenciamento da Tabela de Procedimentos, Medicamentos e Órteses, Próteses e Materiais Especiais do SUS (SIGTAP) que correspondem diretamente às atividades clínicas ampliadas realizadas por enfermeiros. A sequência de buscas, por filtro, realizadas de acordo com o ilustrado na Figura 1 possibilitou a obtenção de um conjunto de dados, os quais foram analisados e relacionados às competências mínimas que caracterizam a PAE, descritas no *Advanced Practice Nursing Characteristics Guidelines on Advanced Practice Nursing 2020* do *International Council of Nurses,* distribuídas em três domínios e 21 itens.

**Figura 1:** Sequência ilustrada de códigos de busca, por filtro, para identificação das atividades realizadas pelos Enfermeiros da Atenção Primária à Saúde, Ribeirão Preto, São Paulo, Brasil

*Fonte: SISAB (2024); ilustração realizada pelos autores.*

Na sessão de resultados, os dados foram organizados em três quadros, em que as competências mínimas do ICN nortearam a indicação de correspondência com as responsabilidades do Enfermeiro, contidas nas diretrizes da PNAB de 2017 do Ministério da Saúde do Brasil e atividades de PAE registradas no SISAB. Esses códigos foram organizados em uma tabela, para compor a terceira coluna, permitindo identificar e categorizar as ações específicas realizadas por esses profissionais no âmbito da AB.

Na etapa de mapeamento dos códigos presentes na plataforma de relatórios do SISAB, foram utilizados filtros específicos para realizar a extração de dados nas categorias Saúde/Produção e Saúde/ Atividade Coletiva (Figura 1).

### Análise dos dados

Para identificar e categorizar as ações específicas realizadas pelos enfermeiros no âmbito da APS, foi realizado o mapeamento dos códigos presentes na plataforma de relatórios do SISAB, nas categorias Saúde/Produção e Saúde/Atividade Coletiva.

Foram considerados procedimentos e atendimentos existentes na tabela unificada do SIGTAP que correspondem diretamente às atividades clínicas ampliadas realizadas por enfermeiros. A sequência de buscas, por filtro (realizadas de acordo com o ilustrado na Figura 1), possibilitou a obtenção dos dados brutos.

A análise comparativa foi empregada nos dados, relacionando-os às competências mínimas que caracterizam a Prática Avançada de Enfermagem, descritas no *Advanced Practice Nursing Characteristics Guidelines on Advanced Practice Nursing 2020* do *International Council of Nurses* do ICN), distribuídas em três domínios, quais sejam: Preparação Educacional, Natureza da Prática e Exercício de Enfermagem de Prática Avançada e 21 itens/características descritos em seus respectivos domínios (Quadros 1, 2 e 3).

**Quadro 1:** Matriz de análise de competências e intervenções de Práticas Avançadas do Enfermeiro na Atenção Primária à Saúde, classificadas segundo o domínio *Preparação Educacional*, de acordo com as diretrizes do *International Council of Nurses*, da Política Nacional de Atenção Básica e do Ministério da Saúde do Brasil, Ribeirão Preto, São Paulo, Brasil

Preparação Educacional		
*International Council of Nurses*, 2020	Política Nacional de Atenção Básica 2017	Sistema de Informação em Saúde para a Atenção Básica 2023
1. Preparação educacional adicional à formação como enfermeiro generalista ou especialista, com requisito mínimo de um programa de mestrado completo (módulos de nível de mestrado tomados como cursos separados não atendem a esse requisito).	Enfermeiro, preferencialmente especialista em saúde da família (atualmente, diverge do *International Council of Nurses*, que recomenda no mínimo o mestrado em Prática Avançada de Enfermagem.	Indisponível
2. Reconhecimento formal de programas educacionais que preparam enfermeiros especificamente para Enfermagem de Prática Avançada (por exemplo, credenciamento, aprovação ou autorização por órgãos governamentais ou não governamentais).	CAPÍTULO I DAS RESPONSABILIDADES VII - Articular, com o Ministério da Educação, estratégias de indução às mudanças curriculares nos cursos de graduação e pós-graduação na área da saúde, visando à formação de profissionais e gestores com perfil adequado à Atenção Básica.	Indisponível
3. Um sistema formal de credenciamento vinculado às qualificações educacionais definidas	No Brasil, o credenciamento do enfermeiro da Atenção Primária à Saúde é obtido por meio do Cadastro Nacional de Estabelecimentos de Saúde, após o registro profissional no conselho de classe da categoria.	Indisponível

**Quadro 2:** Matriz de análise de competências e intervenções de Práticas Avançadas do Enfermeiro na Atenção Primária à Saúde, classificadas segundo o domínio *Natureza da Prática,* de acordo com as diretrizes do *International Council of Nurses*, da Política Nacional de Atenção Básica e do Ministério da Saúde do Brasil, Ribeirão Preto, São Paulo, Brasil

Natureza da Prática		
*International Council of Nurses*, 2020	Política Nacional de Atenção Básica 2017	Sistema de Informação em Saúde para a Atenção Básica 2023
4. Função designada para ações concentradas nos cuidados (diretos ou indiretos), prevenção e cura de doenças em serviços de saúde de nível avançado, incluindo cuidados de reabilitação e manejo de doenças crônicas. Essa função está além do escopo da prática de um enfermeiro generalista ou especializado.	4.2. São atribuições específicas dos profissionais das equipes que atuam na Atenção Básica: 4.2.1 - Enfermeiro: I - Realizar atenção à saúde dos indivíduos e das famílias vinculadas às equipes e, quando indicado ou necessário, em todos os ciclos de vida; II - Realizar consulta de enfermagem.	Consultas Problema/Condição Avaliada: Asma, Desnutrição, Diabetes, Doença Pulmonar Obstrutiva Crônica, Hipertensão arterial, Obesidade, Pré-Natal, Puericultura, Puerpério (até 42 dias), Saúde sexual e reprodutiva, Tabagismo, Usuário de álcool, Usuário de outras drogas, Saúde mental, Reabilitação - Doenças Transmissíveis, Dengue, Doenças Sexualmente Transmissíveis, Hanseníase, Tuberculose, Rastreamento de câncer de mama, de colo do útero, Rastreamento do risco cardiovascular.
5. Capacidade de gerenciar episódios completos de cuidados e problemas complexos de saúde, incluindo populações vulneráveis, de acesso limitado e em risco.	II - Realizar consulta de enfermagem, procedimentos, solicitar exames complementares, prescrever medicações conforme protocolos, diretrizes clínicas e terapêuticas ou outras normativas técnicas estabelecidas pelo gestor federal, estadual, municipal ou do Distrito Federal, observadas as disposições legais da profissão; III - Realizar e/ou supervisionar acolhimento com escuta qualificada e classificação de risco, de acordo com protocolos estabelecidos.	Intervenções: Cauterização química de pequenas lesões, Cirurgia de unha (cantoplastia), Cuidado de estomas, Curativo especial, Drenagem de abscesso, Eletrocardiograma, Exame do pé diabético, Exérese/biopsia/punção de tumor, Fundoscopia, Infiltração em cavidade sinovial, Remoção de corpo estranho subcutâneo, Retirada de cerume, Retirada de corpo estranho da cavidade auditiva e nasal, Sutura simples, Inserção de Dispositivo Intra-Uterino, Retirada de Dispositivo Intra-Uterino.
6. Capacidade de integrar pesquisa (prática baseada em evidências), educação, liderança e gestão clínica.	V - Realizar atividades em grupo; VI - Planejar, gerenciar e avaliar as ações desenvolvidas pelos técnicos/auxiliares de enfermagem, Agente Comunitário de Saúde e Agente de Combate às Endemias em conjunto com os outros membros da equipe; VII - Supervisionar as ações do técnico/auxiliar de enfermagem e agente comunitário de saúde; VIII - Implementar e manter atualizados rotinas, protocolos e fluxos relacionados à sua área de competência na Unidade Básica de Saúde; IX - Exercer outras atribuições conforme legislação profissional, e que sejam de responsabilidade na sua área de atuação. CAPÍTULO I DAS RESPONSABILIDADES XVI - Garantir espaços físicos e ambientes adequados para a formação de estudantes e trabalhadores de saúde, para a formação em serviço e para a educação permanente e continuada nas Unidades Básicas de Saúde; Art. 6º Todos os estabelecimentos de saúde que prestem ações e serviços de Atenção Básica, no âmbito do SUS, de acordo com esta portaria, serão denominados Unidades Básicas de Saúde, todas consideradas potenciais espaços de educação, formação de recursos humanos, pesquisa, ensino em serviço, inovação e avaliação tecnológica para a Rede de Atenção à Saúde, constante no seu parágrafo único.; XIV - promover o intercâmbio de experiências entre gestores e entre trabalhadores, por meio de cooperação horizontal, e estimular o desenvolvimento de estudos e pesquisas que busquem o aperfeiçoamento e a disseminação de tecnologias e conhecimentos voltados à Atenção Básica;	Gestão e liderança: Reunião de equipe, Reunião com outras equipes de saúde, Reunião intersetorial, Conselho local de saúde, Controle, Educação em saúde, Atendimento em grupo, Avaliação/procedimento coletivo, Mobilização social e Ações de educação permanente - Temas para Reunião: Questões administrativas, Processos de trabalho, Diagnóstico do território, Monitoramento do território, Planejamento, Monitoramento das ações da equipe, Discussão de caso/projeto terapêutico singular, Educação permanente, Outros
7. Autonomia ampla e expandida (varia de acordo com o contexto do país e cenário clínico).	III - Realizar e/ou supervisionar acolhimento com escuta qualificada e classificação de risco, de acordo com protocolos estabelecidos; (diferentemente do *International Council of Nurses*, é restrita por ser dependente de protocolos).	Autonomia conforme Política Nacional de Atenção Básica: Diabetes, Hipertensão arterial, Pré-Natal, Saúde sexual e reprodutiva, Saúde mental; Doenças transmissíveis, Rastreamento de câncer, Tuberculose, Hanseníase, Sífilis.
8. Gerenciamento de conjunto de casos em um nível avançado	Não é abordado na Política Nacional de Atenção Básica	
9. Habilidades avançadas de avaliação, julgamento, tomada de decisão e raciocínio diagnóstico	II - Realizar consulta de enfermagem, procedimentos, solicitar exames complementares, prescrever medicações conforme protocolos, diretrizes clínicas e terapêuticas ou outras normativas técnicas estabelecidas pelo gestor federal, estadual, municipal ou do Distrito Federal, observadas as disposições legais da profissão; III - Realizar e/ou supervisionar acolhimento com escuta qualificada e classificação de risco, de acordo com protocolos estabelecidos; IX - Exercer outras atribuições conforme legislação profissional, e que sejam de responsabilidade na sua área de atuação;	Consultas de demanda espontânea e atendimentos de urgência: Asma, Desnutrição, Diabetes, Doença Pulmonar Obstrutiva Crônica, Hipertensão arterial, Obesidade, Pré-Natal, Puericultura, Puerpério (até 42 dias), Saúde sexual e reprodutiva, Tabagismo, Usuário de álcool, Usuário de outras drogas, Saúde mental; Reabilitação - Doenças Transmissíveis: Dengue, Doença Sexualmente Transmissível, Hanseníase, Tuberculose; Rastreamento de câncer de mama, de colo do útero; Rastreamento do risco cardiovascular
10. Reconhecidas competências clínicas avançadas que superam aquelas dominadas por um(a) enfermeiro(a) generalista ou especializado(a).	IX - Idem acima	Consulta do enfermeiro com autonomia para avaliação diagnóstica e tratamento, além da execução de procedimentos complexos, tais como: Cauterização química de pequenas lesões, Cirurgia de unha (cantoplastia), Fundoscopia, Exérese/biopsia/punção de tumor, Infiltração em cav. Sinovial, Sutura simples, Inserção de Dispositivo Intra-Uterino, Retirada de Dispositivo Intra-Uterino
11. Capacidade de fornecer serviços de suporte e/ou consultoria a outros profissionais de saúde, enfatizando a colaboração profissional.	V-Realizar atividades em grupo e encaminhar usuários a outros serviços, conforme fluxo estabelecido pela rede local; VI - Planejar, gerenciar e avaliar as ações desenvolvidas por técnicos/auxiliares de enfermagem, agentes comunitários de saúde, agentes de combate a endemias, em conjunto com os outros membros da equipe;	Gestão e liderança: Reunião com outras equipes de saúde, Reunião intersetorial/conselho local de saúde /controle, Mobilização social
12. Planeja, coordena, implementa e avalia ações para aprimorar os serviços de saúde em nível avançado.	VI -Idem acima	Gestão e liderança: Temas para Reunião: Diagnóstico do território / monitoramento do território, Planejamento/monitoramento das ações da equipe
13. É reconhecida como primeiro ponto de contato para clientes e famílias (comumente, mas não exclusivamente, em ambientes de atenção primária à saúde).	I - Realizar atenção à saúde dos indivíduos e das famílias vinculadas às equipes e, quando indicado ou necessário, no domicílio e/ou nos demais espaços comunitários (escolas, associações, entre outros), em todos os ciclos de vida;	Atua de forma compartilhada na carteira de usuários com o médico, intercalando consultas da agenda. Programa Saúde na Escola: Educação, Saúde. Visita domiciliar/institucional por profissional de nível superior; Visita domiciliar por profissional de nível superior

**Quadro 3:** Matriz de análise de competências e intervenções de Práticas Avançadas do Enfermeiro na Atenção Primária à Saúde, classificadas segundo o domínio *Exercício de Enfermagem de Prática Avançada*, de acordo com as diretrizes do *International Council of Nurses*, da Política Nacional de Atenção Básica e do Ministério da Saúde do Brasil, Ribeirão Preto, São Paulo, Brasil

Exercício de Enfermagem de Prática Avançada		
*International Council of Nurses*, 2020	Política Nacional de Atenção Básica 2017	Sistema de Informação em Saúde para a Atenção Básica 2023
14. Autoridade para diagnosticar	II - Realizar consulta de enfermagem, procedimentos, solicitar exames complementares, prescrever medicações conforme protocolos, diretrizes clínicas e terapêuticas ou outras normativas técnicas estabelecidas pelo gestor federal, estadual, municipal ou do Distrito Federal, observadas as disposições legais da profissão;	Pré-Natal, Puericultura, Puerpério (até 42 dias), Saúde sexual e reprodutiva, Tabagismo, Usuário de álcool, Usuário de outras drogas, Saúde mental. Doenças transmissíveis: Dengue; Doenças sexualmente transmissíveis, Hanseníase, Tuberculose, Rastreamento de câncer de mama, de colo do útero, Rastreamento de risco cardiovascular
15. Autoridade para prescrever medicamentos	Idem acima	Classificação Internacional de Atenção Primária/Classificação Internacional de Doenças: Medicação/Prescrição/Renovação/ Injeção
16. Autoridade para prescrever testes diagnósticos e tratamentos terapêuticos	Idem acima	Teste rápido para dosagem de: proteinúria, Vírus da Imunodeficiência Humana, gravidez, hepatite C, sífilis, triagem oftalmológica
17. Autoridade para encaminhar clientes/pacientes para outros serviços e/ou profissionais.	V - Realizar atividades em grupo e encaminhar, quando necessário, usuários a outros serviços, conforme fluxo estabelecido pela rede local;	Conduta: Agendamento para Núcleo Ampliado de Saúde da Família, Agendamento para grupos, Alta do episódio - Encaminhamentos: interno no dia, intersetorial para Centro de Atenção Psicossocial, internação hospitalar, serviço de atenção domiciliar, serviço especializado, urgência - Retorno para: cuidado continuado/programado, consulta agendada
18. Autoridade para admissão e alta de clientes/pacientes em hospitais e outros serviços	Difere do *International Council of Nurses* por não ser regulamentada a autoridade para hospitalização por Enfermeiro no Brasil.	Indisponível
19. Títulos oficialmente reconhecidos para enfermeiros que trabalham como Enfermeiros de Prática Avançada.	Inexistente no Brasil	Indisponível
20. Legislação para conferir e proteger títulos (por exemplo, Enfermeiro(a) Especialista Clínico(a), Enfermeiro(a) Prático(a).	Inexistente no Brasil	Indisponível
21. Legislação e políticas de uma entidade autorizada ou de algum órgão regulatório explícito para Enfermagem de Prática Avançada (por exemplo, certificação, credenciamento ou autorização específica para o contexto do país)	Inexistente no Brasil	Indisponível

Desse modo, a análise baseou-se no documento do ICN supracitado como norteador global da enfermagem, para relacionar as características da Enfermagem de Prática Avançada convergentes com as competências do enfermeiro estabelecidas pela PNAB, segundo as diretrizes para a organização da Atenção Básica, no âmbito do SUS. Assim, procedeu-se à análise integral da referida política, considerando-se as principais responsabilidades atribuídas ao enfermeiro no âmbito da APS, em concordância com os códigos mapeados na plataforma de relatórios do SISAB.

## RESULTADOS

Da análise comparada entre as diretrizes do ICN e da PNAB, resultou a classificação apresentada na terceira coluna do Quadro 1, que revela a indisponibilidade de intervenções correspondentes executadas pelo enfermeiro na APS, registradas no SISAB.

Cotejando-se a correspondência entre diretrizes do ICN e PNAB (Quadro 1), verifica-se que os três itens integrantes da dimensão *preparo profissional* estão presentes na PNAB, mas com requisito inferior ao do ICN, pois exige especialização em Saúde da Família ao invés de Mestrado Profissional em PAE; além disso, embora conste na PNAB a responsabilidade de articulação entre Ministério da Saúde e Ministério da Educação para adoção de estratégias indutoras de mudanças curriculares para formação profissional, com competências de gestão em prol da APS, é de se ressaltar que no Brasil o credenciamento de enfermeiro da APS é obtido por meio do Cadastro Nacional de Estabelecimentos de Saúde (CNES), mediante comprovação de registro profissional no conselho de classe. Diferentemente, o ICN reconhece a acreditação de EPA pela portabilidade de titulação de mestrado em PAE.

Note-se, também, que os três itens que integram a formação profissional não são contemplados pelo Ministério da Saúde em seu sistema de APS.

Em relação ao dez componentes da dimensão “Natureza da Prática” (Quadro 2), a diferenciação essencial está no nível de complexidade, compatível com uma PAE quando, no caso deste estudo, estamos verificando práticas expandidas no nível da APS, o que se constata especialmente nas intervenções contidas no itens cinco, nove e dez do Quadro 2 sobre as atividades de PAE realizadas pelo Enfermeiro na APS, segundo registros de códigos do SISAB no Brasil, em 2023.

Quando comparados os tipos de intervenções de prática avançada previstos nos citados documentos do Brasil em relação aos do ICN (Quadro 3), com exceção dos últimos quatro (18 a 21), que são inexistentes, a documentação brasileira aqui estudada confere autonomia ao enfermeiro para diagnosticar e prescrever medicamentos, testes diagnósticos e tratamentos terapêuticos assim como para encaminhar pacientes para outros serviços e/ou outros profissionais no âmbito da rede.

## DISCUSSÃO

Debates e esforços contínuos dos governos, sob reiteradas recomendações de organizações globais das áreas da saúde e da economia, têm defendido o poder dos cuidados primários para resgatar e transformar os sistemas de saúde e garantir a qualidade de vida de sua população. A APS tem estado no topo da agenda das políticas de saúde global, merecendo investimentos para sua ampliação desde 1978, em Alma-Ata, até Astana, em 2018^(26,27)^.

Em 2008, o Relatório Mundial da Saúde, assinalando o 60º Aniversário da OMS e 30º Aniversário da Declaração de Alma-Ata sobre Cuidados Primários de Saúde, reconhece realidades inaceitáveis e retrocessos evitáveis no desempenho dos sistemas globais de saúde, que evidenciavam um movimento contrário à resposta compreensiva às necessidades de saúde, com iniquidade de acesso, custos elevados e erosão da confiança nos sistemas de saúde, ameaçando a estabilidade social. Assim, o mencionado relatório organizou as reformas dos Cuidados Primários de Saúde, de modo a reorientar os sistemas de saúde em favor de *saúde para todos*; quatro conjuntos de reformas foram adotados:1) reformas de cobertura universal - para mais equidade em saúde, 2) reformas de prestação de serviços - para orientar os sistemas de saúde para as pessoas, 3) reformas da liderança - para autoridades de saúde mais confiáveis e 4) reformas das políticas públicas - para promover e proteger a saúde das comunidades^(28)^.

Nessa perspectiva, o expressivo desafio de se alcançar “Saúde para todos até o ano 2000” deixou de ser consenso e passou a ser visto por instituições financeiras como inalcançável^(27,29)^. Ao longo de 45 anos, os debates foram frequentes na tentativa de implementar políticas e estratégias de saúde. No entanto, metas foram redefinidas e desafios foram sendo sucessivamente identificados, entre os quais, o da escassez de recursos humanos.

A escassez de profissionais de saúde é um problema global que impacta diretamente a qualidade dos serviços e a capacidade dos sistemas de saúde em atender às demandas da população. Esse fenômeno é influenciado por fatores socioeconômicos, políticos e demográficos que variam de acordo com o contexto de cada país ou região. Estima-se que, até 2030, o mundo enfrentará um *déficit* de cerca de 10 milhões de profissionais de saúde, principalmente em países de baixa e média renda. Esse *déficit* afeta, sobretudo, enfermeiros, médicos e agentes comunitários de saúde. A insuficiência de profissionais da saúde gera impactos negativos em diversas áreas, como limitação no acesso aos serviços, tempo de espera para atendimento, cobertura limitada dos serviços, sobrecarga de trabalho dos profissionais. As estratégias para reduzir o *déficit* concentram-se na expansão da formação profissional^(9)^ e na capacitação contínua do capital humano ao longo da vida laboral, fonte real para a implementação de mudanças^(30)^.

O relatório sobre o estado da Enfermagem de 2020^(9)^ trouxe informações mais recentes sobre como melhorar a força de trabalho de enfermagem em todo o mundo, destacando o valor profissional e apontando o que precisa ser aprimorado para melhorar os serviços de saúde e atingir metas globais; sobretudo, ressaltou a necessidade de investir de maneira considerável em: a) educação dos enfermeiros, garantindo formação de qualidade; b) geração de empregos, oferecendo melhores condições de trabalho; c) desenvolvimento de lideranças na área, para que os enfermeiros possam influenciar políticas de saúde.

Organizações internacionais de enfermagem, consultores e pesquisadores impulsionaram politicamente o debate^(31-36)^, alguns deles reiterando que a subutilização do preparo profissional dos enfermeiros^(34,37-39)^ precisa ser definitivamente superada por uma política que invista nesses profissionais, garantindo que eles possam atuar com seu máximo potencial e, assim, dar substancial contribuição para melhorar a saúde global e atingir metas de desenvolvimento sustentável. Sem a atuação dos enfermeiros, não haverá sustentabilidade de desenvolvimento e nem cobertura universal de saúde para todos, em todos os lugares^(36)^. O relatório detém também a força política capaz de transformar o chamado em ação, uma vez que conclama os Estados-Membros e demais interessados a se comprometerem com essa agenda de investimentos nos enfermeiros, destacando que esses recursos humanos não só impulsionarão a saúde mas também contribuirão para avanços em educação, igualdade de gênero, trabalho decente e crescimento econômico^(9)^.

Desse modo, se previamente já havia ações e evidências de resultados positivos e reconhecimento sobre a tão necessária expansão do escopo da prática conferindo-se mais autonomia para enfermeiros, após o chamado de ação em prol do investimento na enfermagem por instâncias internacionais, envolvendo ministérios da saúde de 191 países, tem-se que a PAE consiste em estratégia vital e altamente eficaz para reduzir a escassez e propiciar o alcance da cobertura universal de saúde.

O ICN considera o poder da enfermagem para assumir uma prática avançada de enfermagem^(40)^, determinando diretrizes e oferecendo conceitos que possam ser adotados por formuladores de políticas que contemplem o aproveitamento de todo o potencial dos enfermeiros, nos âmbitos de governo, educação, sistemas de saúde e de prática profissional, para apoiar iniciativas de prática avançada de enfermagem. Nesse entendimento é que se justificam coerência e clareza na concepção de uma enfermagem capaz de atender às necessidades de saúde das pessoas e dos sistemas de saúde, globalmente^(7)^.

A PAE se distingue da prática tradicional de enfermagem pelo seu nível de especialização e autonomia profissional, permitindo que o enfermeiro realize intervenções complexas, prescreva medicamentos (em alguns contextos), solicite exames diagnósticos e desenvolva planos terapêuticos. Além disso, esses profissionais desempenham um papel fundamental na educação em saúde, gestão de equipes e formulação de políticas públicas.

Analogamente, a convergência entre a PNAB e ICN, vista nos itens 5, 6, 7, 9, 12 e 13 deste estudo, vem ao encontro dos achados na literatura científica recente^(41-43)^ ao descreverem a autonomia profissional do Enfermeiro na APS com respaldo em protocolos assistenciais, os quais garantem clínica ampliada, tomada de decisão, aumento da resolutividade centrada nas necessidades do usuário, continuidade do cuidado, construção de vínculos com a comunidade, autonomia nas áreas de gestão/gerência de práticas individuais e coletivas voltadas à prevenção.

Da mesma forma, no domínio Natureza da Prática e Exercício de Enfermagem de Prática Avançada, itens 4, 7, 14, 15 e 16, o Enfermeiro da APS, no Brasil, destaca-se pela autonomia clínica para diagnosticar, prescrever medicamentos, testes diagnósticos e tratamentos terapêuticos a pessoas com Diabetes, Hipertensão arterial, no Pré-Natal, na Saúde sexual e reprodutiva, Saúde mental, nas Doenças Transmissíveis, no Rastreamento de câncer, Tuberculose, Hanseníase e Sífilis gestacional.

Em 2021, ações de PAE no contexto da Estratégia Saúde da Família foram mapeadas, destacando a autoridade do enfermeiro na solicitação de exames, como análises clínicas citopatológicas, mamografia de rastreamento, hemograma, glicemia de jejum, VDRL, HIV, HBsAg, urocultura, antibiograma, sorologia para toxoplasmose, exames laboratoriais e de imagem, além de exames de fezes, urina, ultrassonografia obstétrica, entre outros exames específicos do pré-natal, saúde da mulher e da criança. O mesmo estudo também detectou autonomia para a prescrição de medicamentos, como ácido fólico, sulfato ferroso, nistatina, miconazol, fluconazol, paracetamol, antibióticos e outros medicamentos disponibilizados no protocolo de enfermagem do município estudado^(17)^. Na mesma direção, em 2022, foi apontada, em outro estudo, a frequência da consulta de enfermagem bem como a solicitação de exames, como hemograma e outros de sangue, ultrassonografia assim como prescrição de medicamentos, sendo os mais prescritos o sulfato ferroso e outros suplementos^(44)^.

De maneira idêntica, nosso estudo demonstra, nos Itens 5 e 10, atuação autônoma do enfermeiro para avaliação diagnóstica e tratamento, além da execução de procedimentos complexos, tais como; sutura simples, fundoscopia, cauterização química de pequenas lesões, inserção de Dispositivo Intra-Uterino (DIU) e outros. Ressalte-se que, em 2023, a realização de sutura simples pelo enfermeiro foi regulamentada pelo Cofen^(45)^.

Após habilitação de enfermeiros para inserção de DIU, em 2018, verificou-se que, até maio de 2021, foram registradas 2.024 inserções realizadas por esses profissionais^(46)^ somente no estado de Santa Catarina. No âmbito do país, nesse mesmo ano, os enfermeiros realizaram 4.653 inserções de DIU, enquanto os médicos realizaram 13.590^(47)^. Em contrapartida, foi observado o número de 54.186 consultas de planejamento reprodutivo, sendo 41.184 (76%) pelo enfermeiro e 13.002 (24%) pelos médicos. Esses achados destacam a importância do enfermeiro na ampliação do escopo de atuação na APS, evidenciando seu papel fundamental na expansão do acesso aos serviços e na cobertura de saúde.

Evidências como essas, além de outras já registradas na literatura, associadas à matriz de análise produzida neste estudo são demonstrativas de que a PAE, no âmbito da APS, é uma realidade incontestável no Brasil. Assim, é de se esperar que lideranças protagonistas dos setores regulatórios, de políticas educacionais e de saúde, associações profissionais, sociedades científicas e de especialistas da enfermagem, escolas e faculdades transformem o chamado em ação, nos respectivos setores, de modo que as evidências aqui registradas sejam revertidas em ações para mudança.

Conforme bem salientado, os países que desejam aumentar o escopo de prática de enfermeiros e o fortalecimento da PAE para melhoria do acesso e da cobertura em saúde devem garantir políticas de regulação, normatização, remuneração, financiamento e formação adequadas a esses enfermeiros capazes de fortalecer a qualidade do sistema de saúde, reduzir custos, valorizar os enfermeiros, favorecendo a resolutividade das demandas de assistência à saúde e impulsionando a equidade^(48)^.

Não obstante, o Brasil ainda carece de amparo deontológico que garanta, para além da legislação vigente, possibilidades para que o enfermeiro exerça, com autonomia, a PAE, uma vez que ainda são presentes as barreiras normativas e culturais que distanciam o enfermeiro de suas competências às demandas de saúde da população, como no caso de solicitação de exames, prescrição de medicamentos protocolados e encaminhamentos, entre outras, na APS.

Ademais, a matriz de análise aqui sinalizada representa uma estrutura conceitual comparativa e demonstrativa da evidência de práticas avançadas que vêm sendo assumidas e registradas pelos enfermeiros no sistema de dados do SUS, com acesso aberto.

### Limitações do estudo

A natureza documental e exploratória do estudo revela potenciais limitações, especialmente ao se restringir à análise de dados secundários, sem observação direta das práticas avançadas realizadas por enfermeiros. Ademais, o uso exclusivo do SISAB pode apresentar lacunas de informação, uma vez que os dados dependem da qualidade do registro realizado por cada município brasileiro, cuja abrangência, em se tratando de dados nacionais, não garante homogeneidade nos registros entre as diferentes regiões do país, embora seja esse o melhor indicador possível para um estudo de caráter documental.

### Contribuições para a área da Enfermagem

A matriz desenvolvida neste estudo representa uma ferramenta estratégica de gestão, e a usabilidade da sequência ilustrada de códigos de busca, por filtro, para identificação das atividades realizadas pelos Enfermeiros da APS, permite aos interessados obter dados de atividades do Enfermeiro no âmbito da APS e, assim, conhecer a amplitude do escopo da prática profissional.

## CONCLUSÕES

A matriz de análise permitiu categorizar as atividades de Práticas Avançadas de Enfermagem no Brasil, de modo a evidenciar os progressos e limitações no contexto da APS, em um país de dimensões continentais, que celebra e reitera o acesso universal à saúde a partir do SUS.

No estudo, observou-se que, embora os enfermeiros possuam competências que conferem autonomia para práticas importantes, como prescrição de medicamentos, solicitação de exames e encaminhamento de pacientes, as políticas brasileiras apresentam lacunas em relação às recomendações internacionais do ICN e da própria OMS.

A exigência de especialização em Saúde da Família, em vez de mestrado em PAE, por exemplo, evidencia certo descompasso na formação profissional, de modo a comprometer a padronização da qualificação exigida para o enfermeiro, nesse nível de formação e intervenção, para a APS. Ademais, a estrutura regulatória do Brasil contempla integralmente os componentes da “Natureza da Prática” de PAE, limitando a expansão dessas atividades na própria APS. Entretanto, tal realidade deve ser objeto de debates e reflexões sobre as demandas e necessidades da saúde e da enfermagem brasileira.

## Data Availability

Os dados de pesquisa estão disponíveis em repositório: https://www.medrxiv.org/content/10.1101/2025.04.30.25326567v3.full.pdf+html.
